# Better Later Than Never: Meaning in Late Life

**DOI:** 10.3389/fpsyg.2021.693116

**Published:** 2021-07-02

**Authors:** Nancy A. Pachana, Roy F. Baumeister

**Affiliations:** School of Psychology, The University of Queensland, Brisbane, QLD, Australia

**Keywords:** meaning, purpose, value, aging, late life, mattering

## Abstract

The quest for meaning in life takes on new challenges and directions during late life. This mini-review draws on prior theory to analyze meaningfulness into six discrete dimensions (purpose, value, efficacy, self-worth, mattering, and comprehension) and covers research into how these apply and operate specifically during late life. Limited remaining time, concern with one's legacy, concerns with self-continuity and integration, variable challenges to self-worth, and prioritization of positivity emerge as key themes.

Finding meaning in life is a potential concern for people of all ages, presumably except very young children. Meaning in life is widely regarded as an indicator of well-being (Ryff, 1989), and indeed most people generally report that their lives have sufficient meaning (Heintzelman and King, 2014). Nevertheless, a lack of meaning is often linked to multiple negative outcomes, again at all ages. For example, lack of meaningful life engagement has been associated with both state and trait loneliness in younger people (Tam and Chan, 2019) and with increased risk of depression in older people (Van der Heyden et al., 2015).

Later life has been mooted as a time of particular relevance to meaningfulness concerns. Later life presents relatively few new opportunities for meaning while taking away some sources that may have been important during youth and middle age. In particular, the loss of social connection due to death or departure of loved ones can reduce meaning, given the central importance of relationships (e.g., Stillman et al., 2009). Yet many researchers, and older adults themselves, would dispute a lack of meaning in later years (Wong, 1998). For example, Reichstadt et al. (2010) found engagement with life and self-growth in later life cited by older adults as key to successful aging (along with self-acceptance and self-contentedness). Early work comparing researchers' and older adults' views of successful aging highlighted the multidimensional formulations of aging well among the latter compared with the former (Phelan et al., 2004). Increasingly, researchers are taking a more nuanced view of successful aging to incorporate constructs including meaningful engagement in life, and moreover taking cultural frameworks into consideration when examining meaning in later life (e.g., Lamb, 2014).

Two influential theories seek to explain how developmental stage of life influences perceptions and behaviors regarding meaning over the life course. Carstensen's Socioemotional Selectivity Theory (SST; Carstensen et al., 1999) suggests that recognition of a foreshortened future influences goal selection such that emotionally meaningful goals and relationships are prioritized over less meaningful goals. Whereas, the young person may see inchoate decades stretching ahead, the older individual is often increasingly aware that time is limited. The time perspective, rather than chronological age *per se*, drives these changing motivational patterns; for example, in younger HIV patients with short life expectancies, similar motivational shifts occur (Carstensen and Fredrickson, 1998). There is a greater urgency to set realistic goals and pursue them without delay. Learning is less important, whereas enjoying meaningful goals gains in priority.

Baltes's Selection Optimization Compensation (SOC) is a theory developed to explain successful aging (Freund and Baltes, 1998) and has been applied in contexts as varied as business, health care, and nursing home settings. SOC focuses not on outcomes but on the processes to maximize gains and minimize loss in pursuit of meaningful goals in later life by harnessing strengths and strategically using adaptive strategies. The SOC model proposes that well-being and effective functioning throughout the lifespan can be achieved by engaging in the strategies of selection (selecting and prioritizing goals), optimization (acquiring and applying means for goal achievement), and compensation (increasing effort or recruiting alternative means to achieve the goal in the face of declines or losses of functioning). Therefore, aging well involves behavioral adjustments but moreover, requires an evolving skill set that includes letting go of plans and procedures developed earlier in life but less suited to current circumstances.

In this brief review, we discuss the challenges that arise in old age with regard to meaning in life. The analysis rests on several prior treatments as to what constitutes meaning in life, starting with Frankl's 1959 seminal work and including subsequent efforts. Frankl focused on purpose as essential to life's meaning. Baumeister (1991) proposed purpose, value, efficacy, and self-worth as four needs for meaning. George and Park (2016) featured purpose, mattering, and comprehension.

## Purpose

All theories about meaning in life emphasize purpose, and it was the main need emphasized in the seminal work by Frankl (1959). Purpose means that present activities draw meaning from future events, to which they are usually connected in causal fashion. Goal pursuit connects present action to future benefits. In short, all meanings of life include purposiveness.

Purposiveness changes with aging. The foreshortened future focuses attention on shorter-term goals—or else one accepts that one is working toward goals that one will never live to see realized. That latter is a bit of a shock, as it goes beyond what animals could consider as worth doing. It leads to thoughts of one's legacy; people begin to care about how they will be remembered after they are dead, though there is no rational benefit to the self (e.g., Vonasch et al., 2018). In a study of legacy, Newton et al. (2020) found that Erikson's construct of stagnation (the opposite of generativity, or caring for the next generation) inhibited legacy expression and well-being in later life.

## Value

Values are beliefs about what is right and good. They guide goal selection. Perhaps there is no strong reason why values should change with aging, but priorities among one's values may change, again because of the foreshortened future, as well as other reasons. If nothing else, aging individuals may become less willing to invest time and effort based on other people's foolish or dubious pursuits. Continuation of midlife generativity may depend on perceptions of whether one's efforts have been respected and appreciated (Cheng, 2009). Still, generativity becomes a prominent value during mid-life and continues into old age for many people (McAdams et al., 1993).

Here an aspect of Cartensen's SST may be relevant: the positivity effect. SST posits that people place increasing value on emotionally meaningful goals as they age. However, older adults also show preferential cognitive processing of positively-valenced (relative to negatively-valenced or neutral) stimuli, compared with younger adults (Charles et al., 2003). Such an emotionally gratifying focus may enable older adults to view life as more positive, and potentially more meaningful, contributing to emotional well-being (Carstensen and Mikels, 2005).

## Efficacy

As a core need for meaning in life, efficacy means making a difference in the world, and especially to bring about progress toward those valued goals. Efficacy varies greatly among persons of any age; later in life it may well be that beliefs about efficacy, that is, self-efficacy and perceived control, may influence well-being and functionality more than efficacy *per se* (Seeman et al., 1996; Robinson and Lachman, 2017). Furthermore, both SST and the positivity effect would argue that one's view of efficacy itself shifts in the latter half of life, such that emotionally gratifying pursuits are prioritized.

## Self-Worth

There is a mixed literature regarding changes in self-esteem with increasing age; while some research supports declines in self-esteem in later life (e.g., Orth et al., 2010), a recent systematic review of the literature spanning 48 countries has found that both age and gender influence self-esteem and self-worth in complex, often non-linear ways (Bleidorn et al., 2016). Complex mediational relationships have also been demonstrated between optimism and self-esteem over the lifespan, with greater optimism associated with increased age (Jiménez et al., 2017). Given an increase in individual differences with increased age, it may well be that while a decline in self-worth is not inevitable in later life, such declines may be experienced more acutely in subpopulations of older adults. This appears to be borne out by converging data on the negative impact of poor health and socioeconomic status on self-esteem in later life (McMullin and Cairney, 2004).

Self-esteem plays an important role in buffering the effects of declines in health and functionality in later life (Sargent-Cox et al., 2012). One salient threat to self-esteem and self-worth in later life is ageism. Levy and Macdonald's 2016 review of the topic highlights negative personal and societal effects of ageism, including age stereotyping and age discrimination in the workplace. Stereotype embodiment theory (Levy, 2009) focuses on individuals who may embody or who have internalized stereotypes of aging in self-fulfilling ways that influence their health. Ravary et al. (2020) have introduced the construct of age-contingent self-worth to disentangle insecurities about one's own aging and feelings of self-worth. Such individuals are argued to be at greater risk for less successful aging, being essentially deprived of the dividends of the positivity effect.

## Mattering

Mattering was proposed by George and Park (2016) as an important component of meaning in life. Mattering, or the belief that one's life matters, is not a well-defined term, though neither is meaningfulness. George and Park explained that mattering involves a subjective belief that one's life has value and significance in the world. Value has already been discussed as a separate need, though for George and Park, value mainly modifies other needs. (They agree that purpose is an important need for meaning, but they also acknowledge that purposes must have value).

The concept of mattering calls for further elucidation. Clearly the term resonates, because self-rated mattering among workers in organizations predicted a variety of positive outcomes (Reece et al., 2021). Mattering has even become a political slogan, with many Americans marching to support the view that “Black lives matter” and some people losing their jobs for suggesting that other lives matter too. In meaning research, however, mattering is used more broadly, such that all lives may contain any degree of mattering.

It may be difficult to sustain a sense that one's own life matters if one does not believe one's life matters to others. Taylor and Turner (2001) discuss mattering in terms of its (negative) relationship to depressive symptomatology (found in their studies in women but not in men) but further work on mattering, effective disorders and suicide, which is a significant issue in later life (particularly for older men; Van Orden and Conwell, 2011) is warranted.

## Comprehension

Comprehension was the third need postulated by George and Park (2016). It comprises coherence and understanding, so again this need can be broken down into discrete parts. Coherence means that the parts of one's life fit together without contradiction; understanding means that one's life, upon reflection, makes sense.

There is an increasing literature examining age differences across the lifespan in self-continuity, defined as perceived associations of one's present self with past and future selves (Löckenhoff and Rutt, 2017). Maintaining a sense of continuity in the face of age-related changes is an important factor in maintaining well-being in later life (Baltes et al., 1998), and a growing literature suggests increasing stability in self-continuity with increasing age. Other characteristics of the self also demonstrate age trends toward greater stability in later life, including life philosophies, value systems, personality, and life satisfaction (Terracciano et al., 2005; Lachman et al., 2008; Quoidbach et al., 2013). Older adults themselves are aware of this tendency toward self-stability later in life, and this stability may be driven in part through internal and environmental factors (Löckenhoff and Rutt, 2017). This may in turn impact upon comprehension with respect to one's life toward the end of life.

## Concluding Remarks

As human longevity continues to increase, the keys to successful aging become ever more important. An emerging consensus holds that finding or maintaining meaning is part of successful aging. We have sought here to break meaningfulness down into more precisely defined and specific needs for meaning. Such an approach holds promise for elucidating the problems, challenges, and opportunities for maximizing meaning in late life.

## Author Contributions

NP's expertise on aging combined with RB's expertise on meaning. Ideas emerged from discussion. Both authors wrote parts of the manuscript.

## Conflict of Interest

The authors declare that the research was conducted in the absence of any commercial or financial relationships that could be construed as a potential conflict of interest.
